# Some Facts Concerning Marriage in the Case of Woman

**Published:** 1899-05-27

**Authors:** James Oliver

**Affiliations:** Physician to the Hospital for Women, London


### Hospital Clinics and Medical Progress.

### SOME FACTS CONCERNING MARRIAGE IN THE CASE OF WOMAN.

By James Oliver, M.D., F.R.S.Edin., F.L.S., Physician to the Hospital for Women, London.

The subjoined table has been compiled from the records of 1,000 consecutive cases in which the women, when they came under my care, had either reached the menopause, or had been married ten years and had never been pregnant. Occasionally it happens—even when a state of complete sterility has previously been displayed—that a woman brings forth her first child after having lived in wedlock ten years. This, however, is, comparatively speaking, such a rare event that the question of prolificness of marriages cannot be materially affected by these occurrences. It is to be remarked in the first instance that 741, or nearly three-fourths of the 1,000 marriages were contracted during the decade from 18 to 27, both ages inclusive, and that the largest number of these, viz., 113, were celebrated at the age of 20. Aristotle fixes 18 as the most suitable age at which women should marry, but it is noteworthy that at this age a less number (viz., 53) were married than at any other age of our decade. One hundred and seventy-six, or fully one-sixth of the number of marriages, occurred during the second decade, *i.e.*, from 28 to 37; whilst 16 marriages took place during the third decade. Sixty-five marriages were contracted during the ages of 15, 16, and 17. The total number of children begotten of these 1,000 marriages was 4,175, which gives an average of four children from each marriage. A glance at the subjoined table shows that the highest average was yielded by those women who married at the age of 17. It is said that in St. Petersburg four is the average number of children produced by each marriage, but in Switzerland it reaches six. From a careful scrutiny of facts we are justified, however, in asserting that the natural prolificness of women is nearly the same in all countries.

The following table is framed indiscriminately from 1,000 patients (married) seen consecutively:—

| Ages at which Marriage took place. | Respective Numbers Married at Foregoing Ages. | Aggregate Number of Children Produced. | Average Number of Children resulting from each Marriage. |
|---|---|---|---|
| 15 | 10 | 59 | 5·9 |
| 16 | 22 | 100 | 4·5 |
| 17 | 33 | 208 | 6·3 |
| 18 | 53 | 253 | 4·7 |
| 19 | 78 | 438 | 5·6 |
| 20 | 113 | 603 | 5·3 |
| 21 | 76 | 359 | 4·7 |
| 22 | 84 | 350 | 4·1 |
| 23 | 86 | 373 | 4.3 |
| 24 | 59 | 277 | 4·6 |
| 25 | 79 | 346 | 4·3 |
| 26 | 58 | 202 | 3·4 |
| 27 | 55 | 135 | 2·4 |
| 28 | 34 | 142 | 4·1 |
| 29 | 32 | 103 | 3·2 |
| 30 | 31 | 63 | 2·03 |
| 31 | 13 | 49 | 3·7 |
| 32 | 12 | 26 | 2·1 |
| 33 | 12 | 27 | 2·2 |
| 34 | 19 | 23 | 2·2 |
| 35 | 7 | 5 | ·7 |
| 36 | 10 | 15 | 1·5 |
| 37 | 6 | 10 | 1·6 |
| 38 | 4 | 7 | 1·7 |
| 39 | 1 | 0 | 0 |
| 40 | 3 | 1 | ·33 |
| 41 | 5 | 1 | ·2 |
| 42 | 0 | 0 | 0 |
| 43 | 1 | 0 | 0 |
| 44 | 2 | 0 | 0 |
| 49 | 2 | 0 | 0 |
| Totals | 1,000 | 4,175 | 4·175 By each marriage. |

In the above 1,000 cases the menopause had been reached or the woman had been married 10 years and had never been pregnant; 257 had never had a child.

